# Aluminium in human brain tissue from donors without neurodegenerative disease: A comparison with Alzheimer’s disease, multiple sclerosis and autism

**DOI:** 10.1038/s41598-020-64734-6

**Published:** 2020-05-08

**Authors:** Christopher Exley, Elizabeth Clarkson

**Affiliations:** 1 The Birchall Centre, Lennard-Jones Laboratories, Keele University, Staffordshire, ST5 5BG United Kingdom; 2 Wichita State University, 1845 Fairmount Street, Wichita, Kansas 60218-0093 USA

**Keywords:** Neurological disorders, Risk factors

## Abstract

A burgeoning number of studies are demonstrating aluminium in human brain tissue. While research has both quantified and imaged aluminium in human brain tissue in neurodegenerative and neurodevelopmental disease there are few similar data for brain tissue from non-neurologically impaired donors. We have used microwave assisted acid digestion and transversely heated graphite furnace atomic absorption spectrometry to measure aluminium in twenty brains from donors without recognisable neurodegenerative disease. The aluminium content of 191 tissue samples was invariably low with over 80% of tissues having an aluminium content below 1.0 μg/g dry weight of tissue. The data for these control tissues were compared with data (measured using identical procedures) for sporadic Alzheimer’s disease, familial Alzheimer’s disease, autism spectrum disorder and multiple sclerosis. Detailed statistical analyses showed that aluminium was significantly increased in each of these disease groups compared to control tissues. We have confirmed previous conclusions that the aluminium content of brain tissue in Alzheimer’s disease, autism spectrum disorder and multiple sclerosis is significantly elevated. Further research is required to understand the role played by high levels of aluminium in the aetiology of human neurodegenerative and neurodevelopmental disease.

## Introduction

Human exposure to aluminium is burgeoning and its entry into and presence within the human body is inevitable^1–3^. There is no ‘aluminium homeostasis’^4^. The bioinorganic chemistry of aluminium dictates that it will ‘piggy-back’ upon essential biochemistry and it is such adventitious interactions that determine its fate in the human body. The brain is a target tissue for accumulation of aluminium^5,6^. Long-lived neurones provide intracellular pools, such as citrate, ATP and glutamic acid, where aluminium can remain complexed without necessarily disrupting cellular biochemistry^7^. Aluminium is neurotoxic and is found in brain tissue in extracellular milieu associated with neuropathology including senile plaques and neurofibrillary tangles in Alzheimer’s disease^8,9^. While there is no longer any debate as to the presence of aluminium in human brain tissue, there remains the question of how much aluminium in brain tissue is too much^10^. A number of recent studies have provided data on aluminium content in brain tissue in Alzheimer’s disease^11^, multiple sclerosis^12^ and autism^13^. The quantitative data, supported by aluminium-specific imaging, are invariably reported as being high, higher than expected. However, data on the latter, true control data are extremely rare. Brain banks have themselves struggled with the concept of what constitutes a true control^14^. We asked one such brain bank to identify a set of donor brain tissues that could act as a control for brains affected and diagnosed with a neurodegenerative disease. The majority of control brains available through brain banks are from older donors and so most still show some signs of age-related degeneration. Herein we have measured the aluminium content of twenty control brains where in each case there was no overt neurodegeneration, no diagnosis of a neurodegenerative disease but some age-related changes in the older donors. We have then compared these data with data, measured under identical conditions, for donors having died with diagnoses of Alzheimer’s disease, multiple sclerosis and autism.

## Results

### Control brain tissues

**Figure 1:**
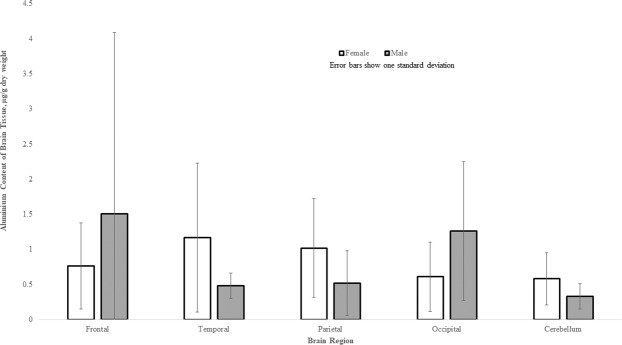
Aluminium content, μg/g dry wt., of brain tissue for each brain region of the twenty donor controls, comprising 5 males and 15 females. Mean and one standard deviation are indicated. For primary data, please see Table 1.

The aluminium content of all tissues ranged from 0.01 (the limit of quantitation) to 9.28 μg/g dry wt. (Table 1). The majority of tissues (150 out of 191) were below 1.00 μg/g dry wt. though 28, 6 and 7 tissues were in the range 1.00–1.99, 2.00–2.99 and ≥3.00 μg/g dry wt. respectively. The aluminium content of each lobe (mean and SD) were 1.03 (1.64), 1.02 (1.27), 0.95 (0.88), 0.77 (0.92) and 0.51 (0.51) μg/g dry wt. for frontal, temporal, parietal, occipital and cerebellum respectively. There were no statistically significant differences between aluminium content and age (p-value = 0.7656) or gender (p-value = 0.4005) and even though the cerebellum had the lowest content of aluminium, there were no statistically significant differences between any of the five brain regions (p-value = 0.2488; Table 2; Fig. 1).

**Table 1:** Aluminium content (μg/g dry wt. tissue) in brain tissues of control donors.

ID	Sex	Age	F	T	P	O	Cb
A002/13	M	90	4.18	0.59	1.06	0.43	0.51
			9.28	0.52	0.92	0.77	Lost
A407/13	F	80	0.85	0.80	0.62	0.43	0.05
			0.01	0.04	0.80	3.62	0.05
A158/14	F	73	0.84	0.27	0.77	1.76	0.30
			0.48	0.32	0.52	0.29	0.82
A132/14	F	66	0.45	0.06	0.62	0.26	1.52
			0.43	0.56	2.35	1.05	0.43
A105/14	F	77	0.85	0.47	0.44	0.34	Lost
			0.82	0.68	5.09	0.11	0.91
A082/14	M	68	0.70	0.20	0.67	1.17	0.85
			0.49	0.46	0.49	4.42	0.10
A177/14	F	67	0.01	1.01	0.29	0.29	0.17
			0.46	4.77	0.44	0.40	0.42
A308/14	F	66	0.43	0.49	1.44	0.48	Lost
			0.38	0.49	0.40	0.29	Lost
A297/14	M	105	0.77	0.61	1.51	2.52	0.04
			0.46	Lost	0.27	0.95	0.20
A007/15	F	74	0.39	0.29	2.19	0.29	0.24
			0.69	0.13	1.73	0.30	0.72
A202/15	F	93	1.25	0.16	1.36	0.49	0.69
			Lost	0.78	0.91	0.75	0.59
A066/16	M	95	0.38	0.43	0.34	1.01	0.21
			1.35	0.57	Lost	0.25	0.55
A006/16	F	68	0.64	0.20	0.74	Lost	0.89
			0.21	2.77	0.72	0.83	0.31
A500/16	F	96	0.40	2.00	0.31	0.35	2.68
			0.52	0.97	0.21	0.24	0.33
A234/17	F	47	1.47	0.58	1.16	0.52	0.20
			0.36	1.30	0.95	1.48	0.76
A103/17	F	55	1.27	1.25	0.73	0.17	0.04
			0.30	1.98	1.38	0.23	0.14
A073/14	F	72	4.72	0.81	1.94	0.16	1.03
			0.83	1.96	1.30	0.32	0.31
A250/14	F	69	0.40	0.97	0.30	0.45	0.45
			0.67	0.92	0.15	1.22	0.61
A242/15	M	82	0.61	0.46	0.61	0.66	Lost
			0.35	0.91	0.62	0.43	0.17
A256/17	F	86	0.11	6.65	0.62	0.18	0.55
			1.49	1.35	0.09	0.15	0.12

F - frontal; T - temporal; P - parietal; O - occipital; Cb - cerebellum - 2 replicate samples for each tissue. Lost - indicates no data due to incomplete digestion of tissue.

**Table 2:** Descriptive statistics for control brain tissues. F - frontal; T - temporal; P - parietal; O - occipital; Cb - cerebellum. Aluminium content in μg/g dry weight of tissue.

Stat/Lobe	F	T	P	O	Cb
Mean	1.03	1.02	0.95	0.77	0.51
Standard Error	0.26	0.20	0.14	0.15	0.09
Median	0.52	0.59	0.73	0.43	0.42
Mode	0.85	0.20	0.62	0.29	0.05
Standard Deviation	1.64	1.27	0.88	0.92	0.51
Sample Variance	2.71	1.62	0.77	0.84	0.26
Kurtosis	17.44	11.10	12.41	7.81	9.18
Skewness	3.96	3.13	3.01	2.71	2.56
Range	9.27	6.61	5.00	4.31	2.64
Minimum	0.01	0.04	0.09	0.11	0.04
Maximum	9.28	6.65	5.09	4.42	2.68
Sum	40.30	39.78	37.06	30.06	17.96
Count	39	39	39	39	35
Confidence Level (95.0%)	0.53	0.41	0.29	0.30	0.17

### Comparison with disease groups

We compared control brain data with each of four treatment groups, namely sporadic Alzheimer’s disease (sAD), familial Alzheimer’s disease (fAD), autism spectrum disorder (ASD) and multiple sclerosis (MS). The descriptive statistics for each of these groups are shown in Table 3. The sAD group is actually composed of both control donors and donors diagnosed with sporadic Alzheimer’s disease, an approximate 50/50 split, see later for a more detailed explanation of this. In addition this group has been analysed according to how negative values in the original data set are dealt with, for example; sAD- sporadic Alzheimer’s disease (including negative values); sAD+- sporadic Alzheimer’s disease (negative values adjusted to LOQ); sAD– - sporadic Alzheimer’s disease (negative values excluded).

**Table 3:** Descriptive statistics for the different groups under comparison.

Stat/Group	Control*	sAD	sAD+	sAD-	ASD	fAD	MS
**Observations**							
Mean	0.95	1.69	1.75	1.95	3.17	2.80	3.20
Median	0.60	1.30	1.03	1.17	1.69	1.41	1.20
Std. Deviation	1.21	2.97	2.91	3.01	4.29	4.58	9.30
Maximum	9.28	32.99	32.99	32.99	22.11	35.65	132.64
Minimum	0.01	−4.91	0.01	0.01	0.01	0.01	0.01
Count	156	708	708	636	56	144	330
**Means for individuals**							
Mean	0.94	1.69	1.75	1.96	3.20	2.80	3.24
Median	0.88	1.31	1.44	1.61	3.56	2.44	2.76
Std. Deviation	0.39	1.05	1.37	1.13	1.41	1.60	1.80
Maximum	2.22	4.74	4.92	5.90	4.77	6.55	6.93
Minimum	0.55	0.37	0.47	0.51	1.20	0.34	1.06
Count	20	60	60	60	5	12	14

Control - control group; sAD- sporadic Alzheimer’s disease (including negative values); sAD+- sporadic Alzheimer’s disease (negative values adjusted to LOQ); sAD–sporadic Alzheimer’s disease (negative values excluded); ASD- autism spectrum disorder; fAD - familial Alzheimer’s disease; MS - multiple sclerosis. Aluminium content in μg/g dry weight of tissue. *Excluding data for cerebellum.

**Figure 2:**
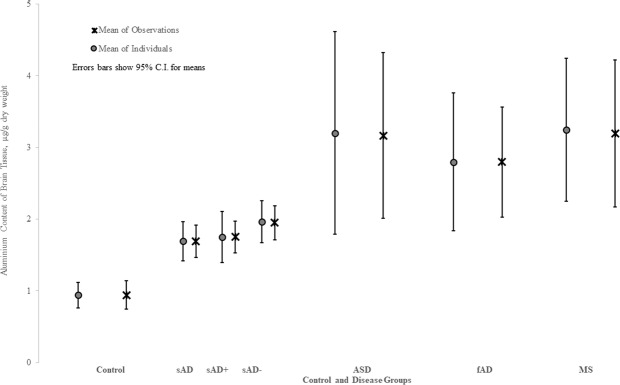
Statistical comparisons, using both mean of observations and mean of individuals, between aluminium content, μg/g dry wt., of brain tissue in control and disease groups. Mean and 95% confidence intervals are indicated. For descriptive statistics, please see Table 3.

Treatment group (sAD; sAD–; sAD + ; fAD; ASD; MS) was the only factor that consistently affected the aluminium content of donor brain tissue. This was the case for all statistical analyses carried out including; when units were unweighted observations or means of individuals; for both adjustments sAD+ and sAD–; for truncation of outliers; parametric or non-parametric procedures (Table 4). The aluminium content of brain tissue in the control group was significantly lower than sAD (P = 0.0006), fAD (P = 0.0020), ASD (P = 0.0123) and MS (P < 0.0001) (Fig. 2).

**Table 4:** Wilcoxon scores, rank sums (Dwass, Steel, Critchlow-Fligner Method) relating to aluminium data (μg/g dry wt.) for unweighted observations and individual means.

Unweighted Observations		
Disease	sAD+n = 1394	sAD-n = 1322
ASD v fAD	0.3912	0.3912
**ASD v Control**	**<0.0001**	**<0.0001**
ASD v sAD	**0.0002**	**0.0068**
ASD v MS	0.0697	0.0697
**fAD v Control**	**<0.0001**	**<0.0001**
fAD v sAD	**0.0043**	0.3970
fAD v MS	0.5880	0.5880
**Control v sAD**	**0.0001**	**<0.0001**
**Control v MS**	**<0.0001**	**<0.0001**
sAD v MS	0.1071	1.0000
**Individual Means**		
**Disease**	**sAD+n = 111**	**sAD-n = 111**
ASD v fAD	0.9171	0.9171
**ASD v Control**	**0.0123**	**0.0123**
ASD v sAD	0.1649	0.2936
ASD v MS	0.9987	0.9987
**fAD v Control**	**0.0020**	**0.0020**
fAD v sAD	0.0956	0.2198
fAD v MS	0.9940	0.9940
**Control v sAD**	**0.0006**	**<0.0001**
**Control v MS**	**<0.0001**	**<0.0001**
sAD v MS	**0.0056**	**0.0347**

Control - control group; sAD- sporadic Alzheimer’s disease (including negative values); sAD+- sporadic Alzheimer’s disease (negative values adjusted to LOQ); sAD–sporadic Alzheimer’s disease (negative values excluded); ASD- autism spectrum disorder; fAD - familial Alzheimer’s disease; MS - multiple sclerosis. Significant P values are shown in bold typescript.

## Discussion

We present the first comprehensive data set for the aluminium content of brain tissue in donors without a diagnosis of neurodegenerative disease. All donors fulfilled recently revised criteria for control brain tissues^14^. Approximately 80% of measured tissues have an aluminium content below 1.0 μg/g dry wt. (Table 1). There are some anomalies, 6 out of 191 tissues have an aluminium content ≥3.00 μg/g dry wt., and these are worth future investigation to identify possible neuropathology. There was no statistically significant relationship between brain aluminium content and age of donor and this observation is contrary to a previous investigation of brain aluminium in a neurologically normal population^15^. An explanation may be that herein only two out of twenty donors were below 66 years old. The data do support a conclusion that a high content of brain aluminium is not an inevitability of ageing.

When we compared the new control data set with data produced in an identical manner in donors dying with diagnoses of sporadic Alzheimer’s disease (sAD)^16^, familial Alzheimer’s disease (fAD)^11^, autism spectrum disorder (ASD)^13^ and multiple sclerosis (MS)^12^ all of these disease groups had significantly higher brain aluminium content. The differences were always highly significant regardless of the method of statistical analysis (Table 4). The largest disease group, designated as sAD, was actually composed of approximately equal numbers of donors previously described by a brain bank as controls and donors diagnosed with sAD. Unfortunately, information discriminating between control and sAD donors was not made available to us^17^. However, the observation that the aluminium content of brain tissue in this group as a whole was significantly higher than the similarly aged control group emphasised the likelihood that brain aluminium content is increased in sAD. The data for the control group demonstrate that high content of brain aluminium is not an inevitability of living in the aluminium age. All disease groups had significantly higher brain aluminium content than the control group in spite of low numbers of donor brains, for example only 5 in ASD, and high variability within measured tissues. The disease groups, sAD, fAD, ASD and MS shared the characteristics of significant focal deposits of aluminium throughout all main lobes of the brain and associated neuropathology and neurodegeneration. Quantitative data, even when complemented with high quality aluminium-specific fluorescence microscopy^18^, do not directly confirm a role for aluminium in each of these diseases. However, since there is no debate as to the neurotoxicity of aluminium in humans^19–21^, such data do implicate aluminium in disease aetiology. Animal models of aluminium intoxication reproduce the neuropathologies and neurodevelopmental effects of human neurodegenerative disease, if not the diseases *per se*^22,23^. Cell models and *in vitro* studies demonstrate mechanisms of aluminium toxicity known to be involved in human neurodegenerative disease^24,25^. Perhaps the information that is still missing from our understanding of aluminium’s role in each of the diseases compared herein is how much aluminium is too much in human brain tissue^10^. The comparison we have made herein between control brain tissue showing no signs of neurodegenerative disease and the disease groups sAD, fAD, ASD and MS is beginning to answer this question. Only further measurements on more donor brains will enable a definitive conclusion to be reached on the role played by aluminium in human neurodegenerative disease.

Aluminium is not a member of the human metallome^4^. However, its omnipresence in human tissue and especially the brain cannot be without consequence. It is only inimical to life, there is no homeostasis, and it is always a burden to life’s processes. Every atom of aluminium in human brain tissue must be accommodated as aluminium as Al^3+^_(aq)_, is highly biologically reactive. Life is robust and some aluminium in human brain tissue is tolerated without overt effects. We need to define such limits in the terms of both quantity and location and we need to be more fully aware of human exposure to aluminium. We may then live healthily in the aluminium age (https://www.hippocraticpost.com/mens-health/the-aluminium-age/).

## Methods

### Tissues

Brain tissues were obtained from the London Neurodegenerative Diseases Brain Bank following ethical approval (NRES Approval No. 08/MRE09/38+5). Donor brains were chosen on our behalf by the consultant neuropathologist at the brain bank. All had a clinical diagnosis of ‘control’ while some had a pathological diagnosis that included age-related changes in tissue. There were five male and fifteen female donors. They were aged between 47 and 105 years old. Tissues were obtained from frontal, occipital, parietal and temporal lobes and cerebellum from all donors.

### Quantitative measurements

The aluminium content of tissues was measured by an established and fully validated method^16^ that herein is described only briefly. Samples of cortex, approximately 1 g in weight, were thawed at room temperature and cut using a stainless steel blade into sections approximately 0.5 g in weight. Tissues were dried for 48 h to a constant weight in an incubator at 37 °C. Dry and thereafter weighed tissues were digested in a microwave (MARS Xpress CEM Microwave Technology Ltd.) in a mixture of 1 mL 15.8 M HNO_3_ (Fisher Analytical Grade) and 1 mL 30% *w/v* H_2_O_2_ (BDH Aristar). Resulting digests were clear with no fatty residues and, upon cooling, were made up to 5 mL volume using ultrapure water (cond. <0.067μS/cm). Total aluminium was measured in each sample by transversely heated graphite furnace atomic absorption spectrometry (TH GFAAS) using matrix-matched standards and an established analytical programme alongside previously validated quality assurance data. The latter included method blanks, detailed descriptions of which have been published recently^10,16^.

### Statistical analyses

#### Comparisons between brain lobes were analysed using control group data

The control group dataset was well balanced with repeated measurements. Analyses were performed using the SAS general linear models (GLM) procedure, including gender, age and lobes as factors. Observations (OB) was the unit of analysis. The programme and raw data are available upon request. We considered a p-value smaller than 0.05 to be statistically significant using the model shown below.

$$\begin{array}{c}<mml:maligngroup xmlns:xlink="http://www.w3.org/1999/xlink"/>Model:\,OB=gender+age+lobe+gender\ast age+gender\ast lobe\\ <mml:maligngroup xmlns:xlink="http://www.w3.org/1999/xlink"/>+lobe\ast age+error\end{array}$$

#### Analysis by disease group

The distribution of aluminium content data is heavily skewed in the treatment groups. Data are not balanced with the number of observations and their respective lobe varying considerably between treatment groups. There is large variability in repeated measurements taken from the same donor. Analyses were performed for both the unweighted observations, and means across all lobes for each individual.

The assumption that the data across all groups are normally distributed, an assumption that underlies any ANOVA model, is questionable at best. A non-parametric approach was used to avoid this assumption. For the non-parametric tests, the SAS NPAR1WAY procedure was used, with the Dwass, Steel, Critchlow-Fligner Method for two-way comparisons between the different disease categories.

Null and Alternative hypothesis

*H*_0_*:: µ*_*i*_ = *µ*_*j*_ *for all i*≠*j (No differences between means of control and disease groups)*

*H*_1_*: µ*_*i*_≠*µ*_*j*_ *for some i*≠*j (At least one difference between means of control and disease groups)*
